# miR‐664a‐5p promotes experimental membranous nephropathy progression through HIPK2/Calpain1/GSα‐mediated autophagy inhibition

**DOI:** 10.1111/jcmm.18074

**Published:** 2024-01-07

**Authors:** Zhiming Shan, Zhenchao Zhuang, Peiyao Ren, Li Zhao, Danna Zheng, Wei Chen, Juan Jin

**Affiliations:** ^1^ Laboratory Medicine Center Zhejiang Provincial People's Hospital (Affiliated People's Hospital, Hangzhou Medical College) Hangzhou Zhejiang China; ^2^ Department of Laboratory Medicine The First Affiliated Hospital of Zhejiang Chinese Medical University (Zhejiang Provincial Hospital of Traditional Chinese Medicine) Hangzhou Zhejiang China; ^3^ Department of Nephrology The First Affiliated Hospital of Zhejiang Chinese Medical University (Zhejiang Provincial Hospital of Traditional Chinese Medicine) Hangzhou Zhejiang China; ^4^ Urology & Nephrology Center, Department of Nephrology Zhejiang Provincial People's Hospital, Affiliated People's Hospital, Hangzhou Medical College Hangzhou Zhejiang China; ^5^ Four Provincial Marginal Traditional Chinese Medicine Hospitals (Quzhou Traditional Chinese Medicine Hospital) Affiliated to Zhejiang University of Traditional Chinese Medicine Quzhou Zhejiang China

**Keywords:** exosomes, membranous nephropathy, miR‐664a‐5p, podocytes, renal tubular epithelial cells

## Abstract

We previously found that miR‐664a‐5p is specifically expressed in urinary exosomes of idiopathic membranous nephropathy (IMN) patients. Homeodomain‐interacting protein kinase 2 (HIPK2), a nuclear serine/threonine kinase, plays an important role in nephropathy. But the function of these factors and their connection in MN are unclear. To investigate the function and mechanism of miR‐664a‐5p in MN, the miR‐664a‐5p expression in HK‐2 cells, exosomes, podocytes and renal tissues were studied, as well as cell growth and apoptosis of these cells, the binding of miR‐664a‐5p to HIPK2 mRNA, the levels of relative proteins and autophagy. The MN progression in MN mice model was also studied. Albumin increased the miR‐664a‐5p content and apoptosis of HK‐2 cells, which was blocked by miR‐664a‐5p antagomir. miR‐664a‐5p bound to the 3′ UTR of HIPK2 mRNA, resulting in the up‐regulation of Calpain1, GSα shear and the inhibition of autophagy level. Autophagy inhibitor CQ blocked the protective effect of miR‐664a‐5p antagomir, HIPK2 overexpression, Calpain inhibitor SJA6017 on albumin‐mediated injury. MiR‐664a‐5p from albumin‐treated HK‐2 cells could be horizontally transported to podocytes through exosomes. Exosomes from albumin‐treated HK‐2 cells promoted progression of MN mice, AAV‐Anti‐miR‐664‐5p (mouse homology miRNA) could improve them. Albumin increases the miR‐664a‐5p level and causes changes of HIPK2/Calpain1/GSα pathway, which leads to autophagy inhibition and apoptosis up‐regulation of renal tubular epithelial cells. miR‐664a‐5p can horizontally enter podocytes through exosomes resulting in podocytes injury. Targeted inhibition of miR‐664a‐5p can reduce the apoptosis of renal tubule cells and podocytes, and may improve the MN progression.

## 1 INTRODUCTION

Membranous nephropathy (MN) is a type of autoimmune disease characterized by diffuse deposition of immune complex under glomerular basal membrane epithelial cells accompanied by diffuse thickening of the basal membrane. Membranous nephropathy has been enlisted as the most common causes of nephrotic syndrome in adults,^1^, ^2^ and the main clinical manifestations include macroalbuminuria, hypoproteinemia, severe edema and hyperlipemia. 30%–40% of patients with MN will progress to end‐stage renal failure after 10–15 years.^3^ Membranous nephropathy is mainly divided into primary MN and secondary MN, and the aetiology of the former is unknown. It is important to reveal the pathogenesis for the early diagnosis and treatment of primary MN.

After injection of cationic serum albumin (cBSA), mice can show a series of MN symptoms.^4^, ^5^ It is reported that albumin treatment can directly induce injury of renal tubular epithelial cells, which closely relates to renal re‐absorption and excretion,^5^ as well as injury of podocytes,^6^ an important component of the glomerular filtration barrier. These results indicate that renal tubular epithelial cells and podocytes are the main target cells in MN model induced by cBSA. MicroRNAs (miRNAs) are a class of endogenous non‐coding RNA about 20–25 nucleotides long. Studies have pointed out that their expression is disordered in kidney tissues of MN patients.^7^, ^8^, ^9^ Exosomes are disc‐like vesicles with a diameter of 30–150 nm secreted by cells,^10^ which are rich in various proteins, lipids and non‐coding RNAs, including miRNAs. Studies show that exosomal miRNAs have multiple biological functions and can regulate the progression of a variety of kidney diseases, including MN,^9^ renal fibrosis^11^ and diabetic nephropathy.^12^ Additionally, studies show that the content of exosomal miR‐199a‐5p secreted by HK‐2 cells, a kind of proximal tubular epithelial cells, increased and can enter the urine, resulting in abnormal miR‐199a‐5p content in the urinary exosome of diabetic nephropathy patients.^13^ It is suggested that the imbalance of urinary exosomal miRNA expression in MN patients may be related to the alteration of miRNA expression profiles in renal tubular epithelial cells. We previously found that compared with healthy volunteers, patients with idiopathic MN (IMN) have unbalanced expression profiles of miRNAs in urinary exosomes and miR‐664a‐5p was the IMN‐specific urinary exosomal miRNA with the largest range of change.^14^ However, the role of miR‐664a‐5p in the progression of MN and cell injury of renal tubular epithelial cells and podocytes remains unclear.

Homeodomain‐interacting protein kinase 2 (HIPK2), which belongs to nuclear serine/threonine kinase, plays an important role in nephropathy. For example, HIPK2 could promote the epithelial‐mesenchymal transition (EMT) process of renal tubular epithelial cells and renal fibrosis through activating the Smad pathway, Notch pathway, NF‐kB pathway and Wnt/β‐catenin pathway.^15^, ^16^, ^17^ The expression of HIPK2 increased in vancomycin‐induced acute kidney injury mouse model and inhibition of HIPK2 could attenuate the vancomycin drove the progression of acute kidney injury to chronic kidney disease.^18^ Apoptosis of normal rat kidney‐52E cells mediated by hypoxia/reoxygenation could be decreased after treatment with HIPK2 knock‐down.^19^ Besides, the expression of HIPK2 could regulated by miRNA, which guides the silencing complex to degrade mRNA or hinder its translation by pairing with the target mRNA. It has been reported that miR‐141 downregulated the expression of HIPK2 via direct interaction with the 3′‐untranslated region of HIPK2 in HK‐2 cells, leading to an inhibition of EMT.^20^ Additionally, miR‐222 or miR‐147b could target HIPK2, resulting in the protection on post‐myocardial infarction cardiac dysfunction^21^ or the progression to pancreatic cancer.^22^, ^23^ But the role of HIPK2 in cBSA‐induced MN model is still unknown, and the connection between miR‐664a‐5p and HIPK2 needs to be investigated.

Autophagy is a process that engulfs and envelops self‐cytoplasmic proteins or organelles into vesicles, fuses with lysosomes to form autophagic lysosomes, and degrades their encased contents, which is generally thought of as a survival mechanism. The protective effects of autophagy has been revealed in MN.^24^ It was reported that HIPK2 can promote autophagy in primary hepatocytes.^25^ Whether the change of autophagy level in MN is regulated by HIPK2 needs to be identified.

Herein, the effect of albumin on the expression of miR‐664a‐5p in HK‐2 cells and podocytes were explored, and the role of miR‐664a‐5p in HK‐2 cell injury and in MN progression was clarified. Mechanically, we found that miR‐664a‐5p decreased the expression of HIPK2 by targeting HIPK2 mRNA, resulting in Calpain1/GSα‐mediated autophagy inhibition, which led to the apoptosis of renal tubular cells. Meanwhile, we found that miR‐664a‐5p could transfer from HK‐2 cells into podocyte through exosomes, resulting in podocytes damage.

## 2 METHODS AND MATERIALS

### 2.1 Cell culture and treatment

HK‐2 cells (procell, CL‐0109, Wuhan, CN), human podocytes (procell, CP‐H075, Wuhan, CN) and HEK293T cells (procell, CL‐0005, Wuhan, CN) were purchased from Wuhan Punocai Life Technology Co., LTD. HK‐2 cells were cultured in MEM medium (procell, PM150410, Wuhan, CN) containing 10% FBS (procell, 164210‐500, Wuhan, CN) and 1% penicillin/streptomycin (procell, PB180120, Wuhan, CN) under 37°C and 5% CO_2_. Podocytes were cultured on Type I collagen (Gibco, 17100‐017, Shanghai, USA)‐coated dishes and cultured in RPMI‐1640 medium (procell, PM150110, Wuhan, CN) containing 20 U/mL IFN‐γ (procell, PCK062, Wuhan, CN) and 10% FBS at 33°C for amplification, and differentiated into mature podiocytes after 14 days of culture at 37°C with RPMI‐1640 medium with 5% FBS but without IFN‐γ. Human embryonic kidney 293 T cells (HEK293T) were cultured in DMEM medium (procell, PM150210, Wuhan, CN) containing 10% FBS and 1% penicillin/streptomycin under 37°C and 5% CO_2_.

### 2.2 qRT‐PCR detection

RNAs were extracted from cells or exosomes by miRNeasy Mini kit (Qiagen, 217,004, Dusseldorf, DE) and reverse transcribed into cDNAs by RevertAid First Strand cDNA Synthesis Kit (Thermo, K1622, Shanghai, CN), then cDNAs were measured by qRT‐PCR with SYBR Green qPCR Master (Roche, 4943914001‐SR, Shanghai, CN), and the amount was calculated by 2^−ΔΔCt^. The primers were as follows: 5′‐TGCGCACTGGCTAGGGAAAATGAT‐3′ (forward) and 5′‐ CCAGTGCAGGGTCCGAGGTATT‐3′ (reverse) for hsa‐miR‐664a‐5p, 5′‐TGCGCTATTCATTTACTCCCCA‐3′ (forward) and 5′‐ CCAGTGCAGGGTCCGAGGTATT‐3′ (reverse) for mmu‐miR‐664‐5p and 5′‐CGCTTCGGCAGCACATATAC‐3′ (forward) and 5′‐AAATATGGAACGCTTCACGA‐3′ (reverse) for U6.

### 2.3 CCK‐8 detection

Cells were transfected with miR‐664a‐5p antagomir (ribobio, miR30005948‐4‐5, Guangzhou, CN) or antagomir NC (ribobio, miR3N0000001‐4‐5, Guangzhou, CN), combined with or without 100 mg/mL albumin^26^ (sigma, A9080, Shanghai, CN) or PBS, or HIPK2 siRNA (5′‐GGUGGAUCCAUCUAGACAA‐3′) or NC siRNA (5′‐CACUGAUUU CAAAUGGUGCUAUU‐3′), or HIPK2 overexpression vector (abmgood, 232980110000, Richmond, Canada) or vector, or 20 μM chloroquine^27^ (autophagy inhibitor, CQ, macklin, C843545, Shanghai, CN) or 100 μM SJA6017^28^ (Calpain inhibitor, MedChemExpress, HY‐118933, Shanghai, CN). After 72 h, CCK8 (MCE, HY‐K0301, Shanghai, CN) solution was added and the OD 450 nm was determined by the plate reader (Molecular Devices Co., Sunnyvale, CA, USA).

### 2.4 Flow cytometry detection

After cell treatment was completed, cells were collected, rinsed twice with PBS, and centrifuged at 1000 rpm for 5 min. Then operation followed the instructions of Annexin V‐FITC/PI cell apoptosis detection kit (JianGSu Keygen Biotech Corp., Ltd., KGA108, Shanghai, CN). After 500 μL Binding Buffer mixed with 5 μL AnnexinV‐FITC and 5 μL PI was added, cells were detected by flow cytometry (CytoFLEX, Beckman, USA).

### 2.5 Double‐luciferase reporter gene assay

The wild‐type and mutated HIPK2 3′‐untranslated regions (3′‐UTR) sequence were, respectively, cloned into pmirGlO Dual‐luciferase miRNA Target Expression Vector (Promega, Madison, WI, USA) to construct HIPK2 3′‐UTR^WT^ and HIPK2 3′‐UTR^MT^. A mixture of Lipofectamine 2000 (Invitrogen, 11668019, Shanghai, CN), double‐luciferase reporter plasmid, and miRNA antagomir NC or miR‐664a‐5p antagomir were transfected into HEK293T cells. Then, the luciferase activity was analysed using a double luciferase reporting system (Beyotime, RG027, Shanghai, CN).

### 2.6 WB detection

Protein samples prepared from cells, exosomes or tissues, were separated by electrophoresis and transferred on PVDF membrane; PVDF membrane was consecutively treated with primary antibodies (HIPK2, Affinity, DF7982, 1:1000, Wuhan, CN; Calpain1, Affinity, DF6306, 1:1000, Wuhan, CN; GSα, sigma, 06‐237, 1:1000, Shanghai, CN; LC3, Affinity, AF5402, 1:1000, Wuhan, CN; P62, Affinity, AF5384, 1:1000, Wuhan, CN; GAPDH, Xianzhi, AB‐P‐R001, 1:1000, Wuhan, CN; CD9, Proteintech, 20597‐1‐AP, 1:1000, Wuhan, CN; TSG101, Affinity, DF8427, 1:1000, Wuhan, CN; CD63, Affinity, AF5117, 1:1000, Wuhan, CN; calnexin, Affinity, AF5362, 1:1000, Wuhan, CN), HRP labelled secondary antibody (HRP labelled secondary antibody of sheep and rabbit, Boster, BA1054, 1:10,000, Wuhan, CN) and ECL reagents. Finally, the film was analysed using Image J software (National Institutes of Health, USA).

### 2.7 Exosome extraction

HK‐2 cells were treated with 100 mg/mL albumin. ECS reagent (exosome extraction and purification kit, umibio, UR52121, Shanghai, CN) was added to the supernatant and the mixture was centrifuged. The precipitation was evenly dispersed with PBS, and the suspension was centrifuged, and the supernatant, rich in exosome particles, was retained. The crude exosomes were transferred into the upper chamber of the EPF column and centrifuged. The liquid at the bottom of the EPF column was collected, which was the purified exosome particles and stored at −80°C.

### 2.8 Electron microscope detection

Samples, ultra‐pure water and PTA dye liquid (sigma, P4006, Shanghai, CN) were dropped on the copper mesh. After cooling, the copper mesh was dried naturally and observed under electron microscope (FEI, Tecnai G20 TWIN, Hillsboro, USA).

### 2.9 Protein concentration detection

BCA protein concentration assay kit (Biyuntian, P0012S, Shanghai, CN) was used to determine exosomal protein concentration. The concentrations were determined by measuring the OD562nm.

### 2.10 Autophagy flux detection

10 μL Ad‐Mcherry‐GFP‐LC3B virus stock solution (Beyotime, C3011, Shanghai, CN) was added to HK‐2 cells. After washing cells with PBS, autophagy flux of cells was observed under confocal laser fluorescence microscope (Olympus, FV3000, Tokyo, Japan).

### 2.11 Exosome uptaken detection

MiR‐664a‐5p with or without Cy3‐labelled was transfected into exosome from HK‐2 cell treated with PBS (Exo^ctrl^), and then 10 μL exosomes was added to human podocytes. After the slide was dried, drops of DAPI (sigma, D9542, Shanghai, CN) dye solution was added, and the slide was sealed and images were observed under a laser confocal fluorescence microscope.

### 2.12 Murine model of MN

The female BALB/c mice were acquired from Yichang University (CN), fed in a 12‐h light/dark cycle chamber at 24 ± 0.5°C. All experimental protocols were approved by the Hospital Institutional Animal Care and Use Committee of Zhejiang Provincial Institute of Traditional Chinese Medicine (Approval No. IACUC‐A202208001) and comply with the National Institutes of Health Guidelines for the Care and Use of Laboratory Animals. A total of 30 female BALB/c mice aged 4–6 weeks were randomly divided into six groups with five mice in each group. The model group was treated with complete Freund's adjuvant (CFA, Sigma, F5881, Shanghai, CN) containing 0.2 mg cationic BSA (cBSA, Chondrex, 9058, Woodinville, USA) for 2 weeks, and then was given intravenous 100 μg cBSA three times a week for 6 weeks.^5^ Mice were treated with intravenous injection of PBS or 100 μg exosomes once a week at the beginning of modelling. Meanwhile, mice were also injected with 1.5 × 10^9^ pfu adeno‐associated virus (AAV‐Anti NC or AAV‐Anti‐miR‐664‐5p) intravenously every 4 weeks.

### 2.13 Urine protein, serum cholesterol and albumin content detection

24‐h urine was collected and urine protein was determined by urine protein quantitative test kit (Nanjing Jiancheng, C035‐2‐1, Nanjing, China) according to OD595nm. Serum cholesterol content was detected by the total cholesterol detection kit (mlbio, ml094953, Shanghai, CN) according to OD550nm. And serum albumin content was detected using the albumin detection kit (mlbio, ml095005, Shanghai, CN) according to OD603nm.

### 2.14 IgG deposition test

After kidney tissue was embedded and sliced, the slices were attached to the slides and baked 60°C. Then, it was successively dewaxed and repaired. Then, the primary antibody (IgG, CST, 4418, 1:100, Shanghai, China) and the second antibody (Goat Anti‐Rat, BIOSS, bs‐0293G‐CY3,1:100, Beijing, CN) were added, then DAPI chromogenic solution was added. The slide was sealed, and the images were observed under a fluorescence microscope (Olympus, IX51, Tokyo, Japan).

### 2.15 Periodic Acid Schiff (PAS) staining

After dewaxed, the slices were soaked in 1% periodic acid solution (Sinopharm, 80098516, Shanghai, China) and washed with distilled water. Schiff's reagent (Sinopharm, 71019654, Shanghai, China) was added, and the slide was incubated at room temperature for 20 min, and the slices were rinsed with water. The slide was treated by Mayer Hematoxylin (sigma, H9627, Shanghai, China) and rinsed until the nuclei turned blue. Then it was observed under a microscope (Olympus, BX53, Tokyo, Japan).

### 2.16 Terminal deoxynucleotidyl transferase‐mediated dUTP‐biotin nick end labeling assay (TUNEL assay)

The slices were treated with protease K working solution from TUNEL cell apoptosis assay kit (promega, G7360, Beijing, CN). 100 μL Equilibration Buffer and TdT enzyme reaction solution was added dropwise successively. Ater termination, 100 μL Streptavidin HRP (streptavidin horseradish peroxidase) solution was added to the slices, and then 100 μL DAB chromogenic solution was added. The slide was sealed after Mayer haematoxylin counterstaining. The slide was observed under a microscope.

### 2.17 IHC detection

Slices were repaired with 0.01 M citric acid buffer (pH 6.0), and then 3% hydrogen peroxide was used to block endogenous peroxidase. Then, the diluted primary antibody (HIPK2, NOVUS, NBP1‐89462, 1:200, Guangzhou, CN) was added and the slices were incubated in wet box overnight. The secondary antibody (Goat Anti‐Rabbit IgG (H + L) HRP, Affinity, S0001, 1:200, Wuhan, CN) was added and the slices were incubated at 37°C for 30 min. Then, the freshly prepared DAB chromogenic solution (biosharp, BL732A, Hefei, CN) was dripped on and the slices were observed under the microscope. The positive signal is brownish yellow or brownish brown. After re‐staining with Harris haematoxylin, the slide was sealed and observed under the microscope.

### 2.18 Data analysis

Statistical software Prism9.0 was used for data analysis, and all data were expressed as Mean ± SD. One‐way analysis of variance or Student's *t*‐test was used for statistical analysis between groups. *p* < 0.05 was considered statistically significant.

## 3 RESULTS

### 3.1 Albumin induces renal tubular epithelial cells apoptosis dependently of miR‐664a‐5p

**FIGURE 1:**
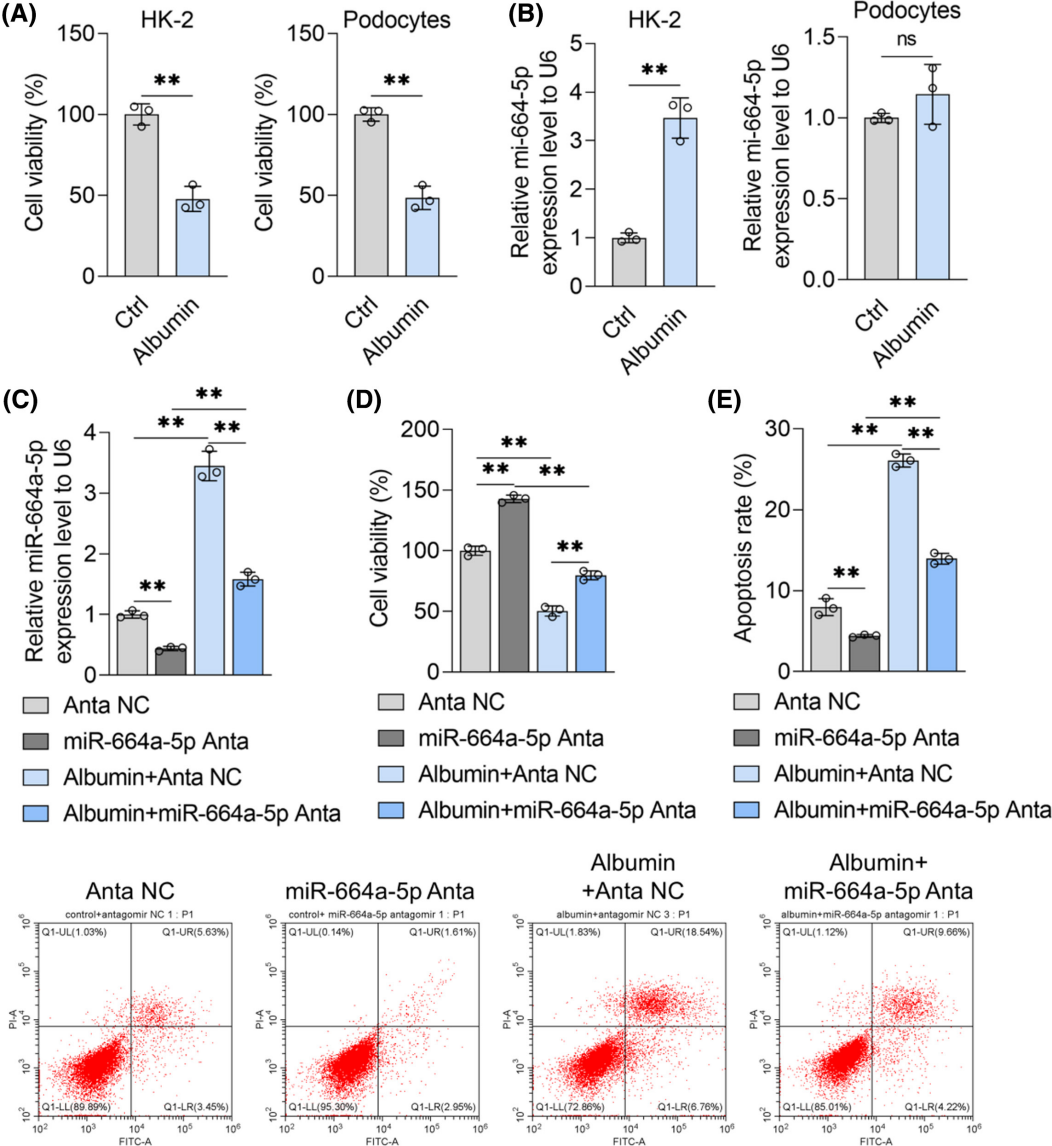
Effects of miR‐664a‐5p antagomir and albumin on HK‐2 cell damage. (A) The cell growth of HK‐2 cells or podocytes after 72‐h treatment with solvents or albumin (100 mg/mL) was measured with the CCK‐8 kit. (B) The intracellular miR‐664a‐5p content of HK‐2 cells or podocytes treated with or without albumin (100 mg/mL) was detected by qRT‐PCR, with U6 as the internal reference. (C–E) HK‐2 cells were treated with a solvent or albumin (100 mg/mL) and transfected with miRNA antagomir NC or miR‐664a‐5p antagomir. After 72 h, qRT‐PCR was used to detect the content of miR‐664a‐5p (C), CCK‐8 assay kit was used to detect the ability of cell growth in each group (D), and flow cytometry with Annexin V‐FITC/PI dye was used to detect apoptosis (E). Data were expressed as Mean ± SD. Student's *t*‐test or One‐way analysis of variance was used for statistical analysis between groups, with ** indicating *p* < 0.01 and ns indicating *p* > 0.05.

HK‐2 cells and podocytes were treated with albumin to construct MN cell models. CCK‐8 assay showed that albumin (100 mg/mL) could reduce the cell growth of HK‐2 cells and podocytes (Figure 1A). It indicated that albumin‐induced cell model of tubular epithelial cells injury and podocytes injury in MN were built successfully. Then, qRT‐PCR experiment showed that albumin treatment increased the content of miR‐664a‐5p in HK‐2 cells rather than podocytes (Figure 1B). Next, miR‐664a‐5p antagomir and albumin was used to co‐treat HK‐2 cells, and results from qRT‐PCR assay showed that the promoting effect of albumin on miR‐664a‐5p content in HK‐2 cells was reversed by miR‐664a‐5p antagomir (Figure 1C). The results of CCK‐8 assay showed that miR‐664a‐5p antagomir treatment increased the cell growth of HK‐2 cells treated without albumin and blocked the inhibitory effects of albumin on the cell viability of HK‐2 cells (Figure 1D). Moreover, flow cytometry with Annexin V‐FITC/PI dye experiment indicated that miR‐664a‐5p antagomir decrease the apoptosis rate of HK‐2 cells in the absence of albumin (Figure 1E). And albumin treatment increased the apoptosis level of HK‐2 cell, which could be inhibited by miR‐664a‐5p antagomir treatment (Figure 1E). It demonstrated that albumin could damage renal tubular epithelial cells, probably depending on miR‐664a‐5p up‐regulation.

### 3.2 miR‐664a‐5p‐mediated HIPK2 expression inhibition was involved in renal tubular epithelial cell injury induced by albumin

**FIGURE 2:**
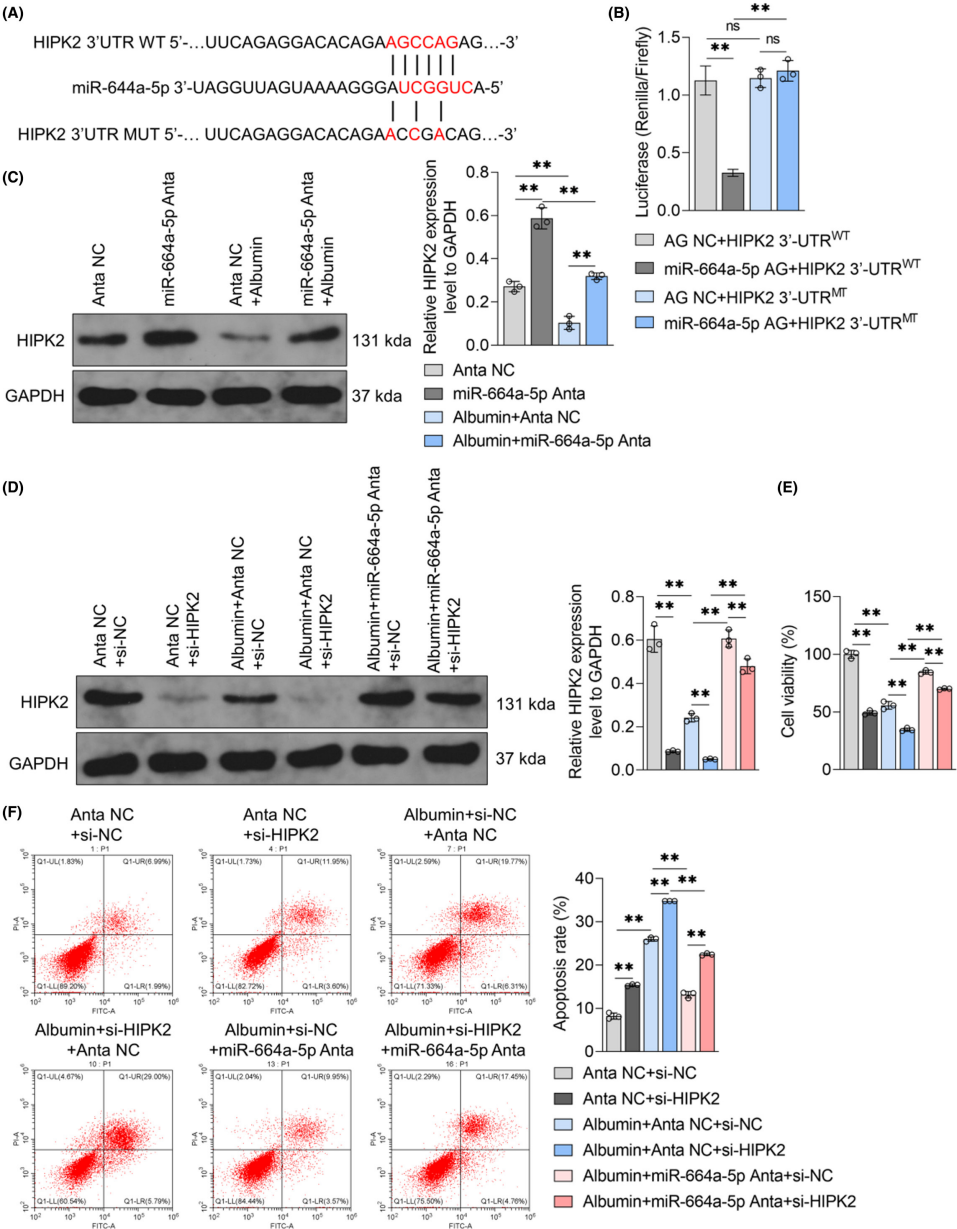
Binding detection of miR‐664a‐5p and the 3′UTR of HIPK2 mRNA and effects of HIPK2 knockdown on HIPK2 expression and damage in HK‐2 cells treated with miR‐664a‐5p antagomir and albumin. (A) The predicted binding site and the mutation sequence of the binding site between miR‐664a‐5p and the HIPK2 mRNA 3′UTR. (B) HEK293T cells were transfected with the luciferase reporter plasmid HIPK2 3′‐UTR^WT^ or HIPK2 3′‐UTR^MT^ toghether with miRNA agomir NC or miR‐664a‐5p agomir, and then the intracellular luciferase activity was detected. (C) Western blot assay was used to detect HIPK2 protein expression in HK‐2 cells treated with solvent or albumin (100 mg/mL) and transfected with miRNA antagomir NC or miR‐664a‐5p antagomir for 72 h. GAPDH was the internal parameter (left panel). The statistical results of grey value of WB strip are shown on the right. (D–F) HK‐2 cells were treated with solvent or albumin (100 mg/mL), combined with miRNA antagomir NC or miR‐664a‐5p antagomir together with NC siRNA or HIPK2 siRNA for 72 h. (D) The intracellular HIPK2 protein content was detected by Western Blot, with GAPDH as the internal reference (left panel). The statistical results of grey value of WB strip are shown on the right. (E) CCK‐8 kit was used to detect the ability of cell growth of HK‐2 cells in each group. (F) Apoptosis was detected by flow cytometry with Annexin V‐FITC/PI dye. Data were expressed as Mean ± SD. One‐way analysis of variance was used for statistical analysis between groups, with ** indicating *p* < 0.01 and ns indicating *p* > 0.05.

**FIGURE 3:**
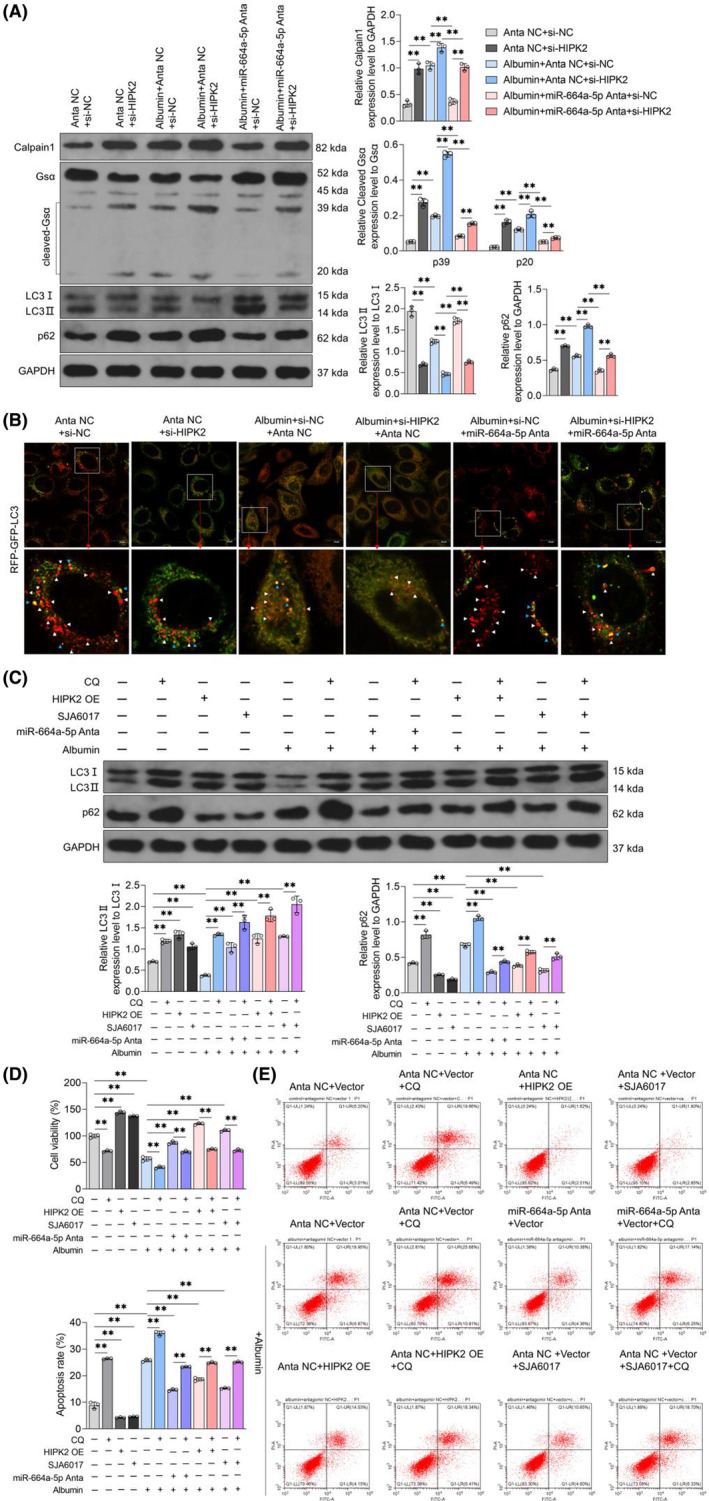
Effects of miR‐664a‐5p/HIPK2/Calpain1/GSα signal pathway‐mediated autophagy inhibition in HK‐2 cells injury caused by albumin. (A) HK‐2 cells treated with solvent or albumin (100 mg/mL) and combined with miRNA antagomir NC or miR‐664a‐5p antagomir. After transfection with NC siRNA or HIPK2 siRNA for 72 h, intracellular Calpain1, GSα shear, LC3II/I ratio and p62 protein content were detected by Western Blot, using GAPDH as reference (left panel). The statistical results of grey value of WB strip are shown on the right. (B) HK‐2 cells were infected with RFP‐GFP‐LC3 adenovirus and treated with solvent or albumin (100 mg/mL) and combined with miRNA antagomir NC or miR‐664a‐5p antagomir in the presence of NC siRNA or HIPK2 siRNA. After 72 h, the changes of autophagy flux in each group were observed under laser confocal microscope. The white arrow indicates autophagic lysosome that has fused with lysosome (GFP^−^RFP^+^ punctate aggregate) and the blue arrow indicates autophagosome that has not fused with lysosome (GFP^+^RFP^+^ punctate aggregate). Scale = 20 μm. (C‐E) HK‐2 cells were treated with solvent or albumin (100 mg/mL), and treated with miR‐664a‐5p antagomir or HIPK2 overexpression or Calpain inhibitor SJA6017 combined with autophagy inhibitor chloroquine meanwhile for 72 h. (C) LC3II/I ratio and p62 protein content in each group were detected by Western blot, using GAPDH as the internal reference. (D) CCK‐8 kit was used to detect the ability of HK‐2 cell growth. (E) Flow cytometry with Annexin V‐FITC/PI dye was used to detect cell apoptosis in each group. Data were expressed as Mean ± SD. One‐way analysis of variance was used for statistical analysis between groups, with ** indicating *p* < 0.01.

miRNAs could modulate gene expression at the post‐transcriptional level either by inhibiting messenger RNA (mRNA) translation or by promoting mRNA degradation. It has been reported that HIPK2 play an important role in kidney disease, including kidney fibrosis,^29^ the progression of acute kidney injury to chronic kidney disease and so on.^18^ Using miRNA target gene prediction database targetscan, binding loci between miR‐664a‐5p and HIPK2 mRNA 3′UTR were predicted and the double‐luciferase reporter containing the wild type sequence or mutated sequence was constructed (Figure 2A). Compared with miRNA agomir NC treatment group, miR‐664a‐5p agomir treatment could reduce the luciferase activity of HIPK2 3′‐UTR^WT^ rather than HIPK2 3′‐UTR^MT^ (Figure 2B). Western blot assay results showed that miR‐664a‐5p antagomir increased HIPK2 protein content in HK‐2 cells and blocked the inhibitory effects of albumin on HIPK2 content in HK‐2 cells (Figure 2C). In addition, HIPK2 siRNA could aggravate albumin‐mediated HIPK2 expression inhibition and prevent miR‐664a‐5p antagomir from raising the HIPK2 protein content in albumin‐treated HK‐2 cells (Figure 2D). In CCK‐8 and flow cytometry experiments, HIPK2 siRNA decreased the cell growth of HK‐2 cells and increased the apoptosis regardless of albumin. MiR‐664a‐5p antagomir blocked albumin‐mediated cell growth inhibition and apoptosis increase of HK‐2 cells, but these effects were inhibited by HIPK2 siRNA (Figure S1A; Figure 2E,F). Moreover, albumin‐mediated cell growth inhibition and apoptosis induction could be blocked by HIPK2 overexpression (Figure S1B; Figure 3D,E). It suggested that HIPK2 expression down‐regulation caused by miR‐664a‐5p was involved in albumin‐mediated renal tubular epithelial cell injury.

### 3.3 miR‐664a‐5p regulated Calpain1/GSα/autophagy signalling pathway through HIPK2 resulting in albumin‐mediated renal tubular epithelial cell injury

It was reported that HIPK2 can promote autophagy, which relates to the expression decrease in Calpain1.^25^ Calpain1 can shear and activate the heterotrimer G‐protein subunit GSα, causing autophagy inhibition in Drosophila.^30^, ^31^ Decreased autophagy is important for the renal tubular cell injury.^32^ We investigated the effects of albumin, miR‐664a‐5p antagomir and HIPK2 on the expression of Calpain1 and GSα shear in HK‐2 cells. The data showed that albumin increased the Calpain1 content, promoted GSα shear and inhibit autophagy level in HK‐2 cells, which were blocked by miR‐664a‐5p antagomir (Figure 3A). Additionally, HIPK2 knock‐down increased Calpain1 expression, GSα shear and decrease autophagy in HK‐2 cells in the presence or absence of albumin and reversed the inhibition of miR‐664a‐5p antagomir on Calpain1 expression, GSα shear and autophagy down‐regulation in HK‐2 cells induced by albumin treatment (Figure 3A,B). Furthermore, the effects of albumin on GSα shear and autophagy was also inhibited by the Calpain inhibitor SJA6017 (Figure S1B; Figure 3C). It demonstrated that miR‐664a‐5p could inhibit autophagy level through HIPK2/Calpain1/GSα signal pathway in renal tubular epithelial cells treated with albumin.

To confirm that autophagy inhibition by miR‐664a‐5p/HIPK2/Calpain1/GSα pathway was related to renal tubular epithelial cell injury, HK‐2 cells were treated with albumin, miR‐664a‐5p antagomir or HIPK2 overexpression or Calpain inhibitor SJA6017 combined with autophagy inhibitor CQ. The data showed that the inhibitory effects of miR‐664a‐5p antagomir, HIPK2 overexpression and SJA6017 on autophagy down‐regulation in HK‐2 cells induced by albumin could be reversed by CQ (Figure 3C). The results of CCK‐8 assay and flow cytometry showed that CQ inhibited cell growth and enhanced apoptosis in HK‐2 cells treated with or without albumin, and miR‐664a‐5p antagomir, HIPK2 overexpression and SJA6017 showed opposite effects, which were blocked by CQ (Figure 3D,E). It suggested that miR‐664a‐5p/HIPK2/Calpain1/GSα signal pathway‐mediated autophagy inhibition was involved in renal tubular epithelial cell injury induced by albumin.

### 3.4 miR‐664a‐5p is transported from renal tubular epithelial cells to podocytes via exosomes

**FIGURE 4:**
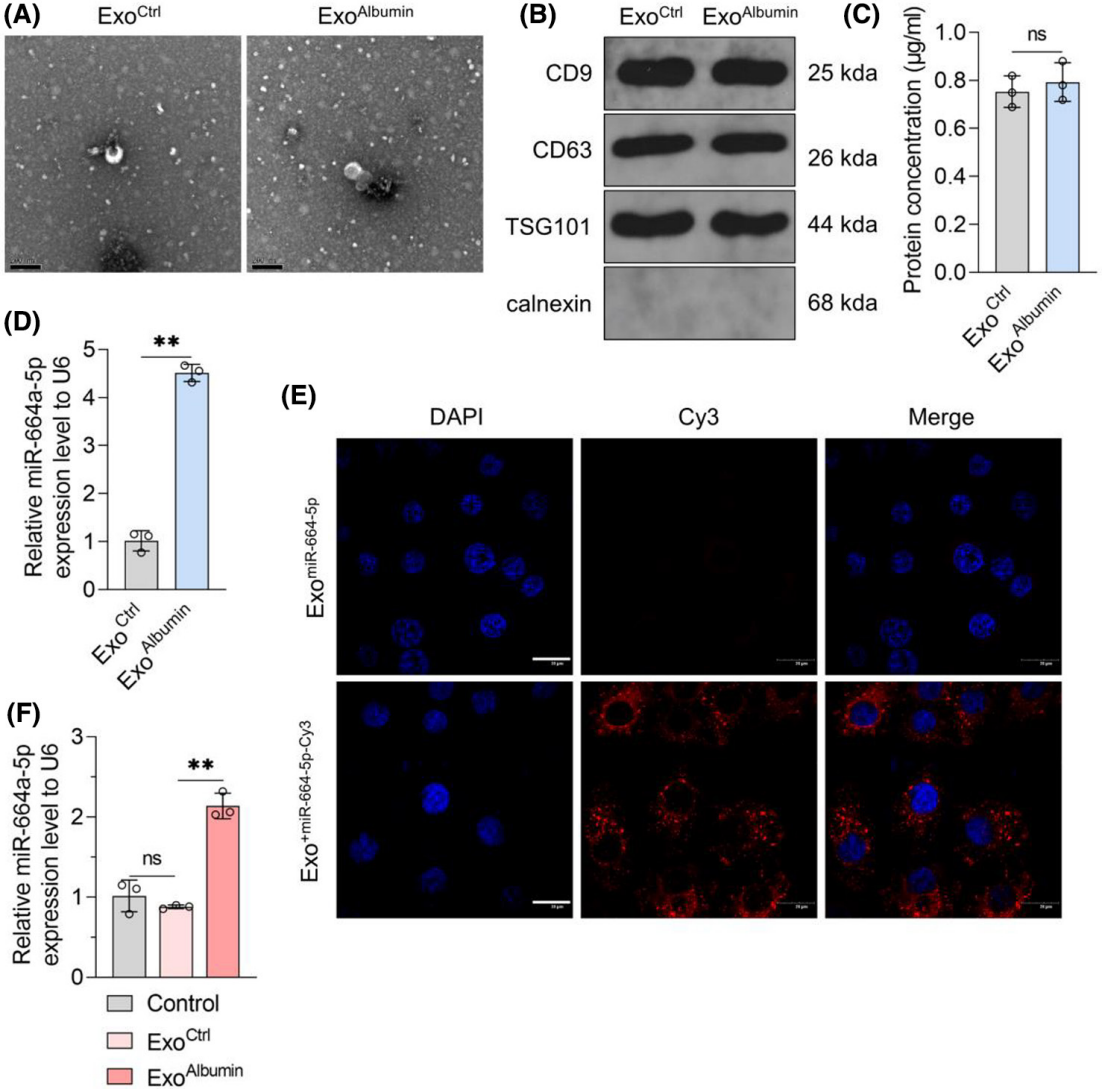
Uptake capacity of renal tubular epithelial cell‐derived exosomal miR‐664a‐5p by podocytes. HK‐2 cells were treated with exosomes‐free FBS, which was produced by high‐speed centrifugation. Then cells were treated with solvents or albumin (100 mg/mL) for 72 h, respectively. Exosomes in the supernatant of cells were collected by exosomes extraction kit (corresponding Exo^Ctrl^, Exo^Albumin^, respectively). (A) Morphology of Exo^Ctrl^ and Exo^Albumin^ was observed under transmission electron microscopy. Scale = 200 nm. (B) The protein expressions of CD9, CD63, TSG101 and calnexin in Exo^Ctrl^ and Exo^Albumin^ were detected by WB. (C) The concentration difference between Exo^Ctrl^ and Exo^Albumin^ between the two groups was detected by the BCA protein concentration assay kit. (D) The content of miR‐664a‐5p in Exo^Ctrl^ and Exo^Albumin^ were detected by qRT‐PCR, with U6 as the internal reference. (E) Cy3‐labelled or unlabeled miR‐664a‐5p agomir was transfected into Exo^Ctrl^, and then co‐incubated with human podocytes for 24 h. The distribution of Cy3 in podocytes of each group was observed under laser confocal microscope. Scale = 20 μm. (F) Effects of Exo^Ctrl^ and Exo^Albumin^ on the content of miR‐664a‐5p in human podocytes were detected by qRT‐PCR, with U6 as the internal reference. Data were presented by Mean ± SD, and Student's *t*‐test or one‐way analysis of variance was used for statistical methods, with ns indicating *p* > 0.05 and ** indicating *p* < 0.01.

Intracellular high‐abundance non‐coding RNA can be secreted into exosomes, and when the exosomes are taken up by target cells, non‐coding RNA in exosomes can be transferred to target cells, achieving horizontal transport of non‐coding RNA.^12^, ^13^, ^33^ It was reported that glomerular endothelial cell‐derived exosomal miR‐192‐5p can reduce the expression of nephronectin in podocytes and participate in the regulation of IMN.^9^ To investigate the exosomal miR‐664a‐5p transport, exosomes from HK‐2 cells was extracted and confirmed by transmission electron microscope and WB (Figure 4A–C). Subsequently, qRT‐PCR assay was used to detect miR‐664a‐5p content in exosomes from HK‐2 cells treated with or without albumin. Data showed that compared with Exo^Ctrl^, the content of miR‐664a‐5p in exosomes from HK‐2 cells treated with albumin (Exo^Albumin^) increased (Figure 4D). Then, Cy3‐labelled miR‐664a‐5p agomir transfected into Exo^Ctrl^ was incubated with podocytes and Cy3 was observed in podocytes under laser confocal microscopy (Figure 4E), indicating that miR‐664a‐5p in exosomes could be taken up by podocytes. Further, qRT‐PCR showed that compared with podocytes without exosome treatment, the miR‐664a‐5p content in podocytes treated with Exo^Ctrl^ did not change and that in podocytes treated with Exo^Albumin^ treatment increased (Figure 4F). These data showed that miR‐664a‐5p from renal tubular epithelial cells treated with albumin could be transported into podocytes via exosomes.

### 3.5 Renal tubular epithelial cells‐derived exosomal miR‐664a‐5p can alter HIPK2/Calpain1/GSα/autophagy pathway and apoptosis of podocytes

**FIGURE 5:**
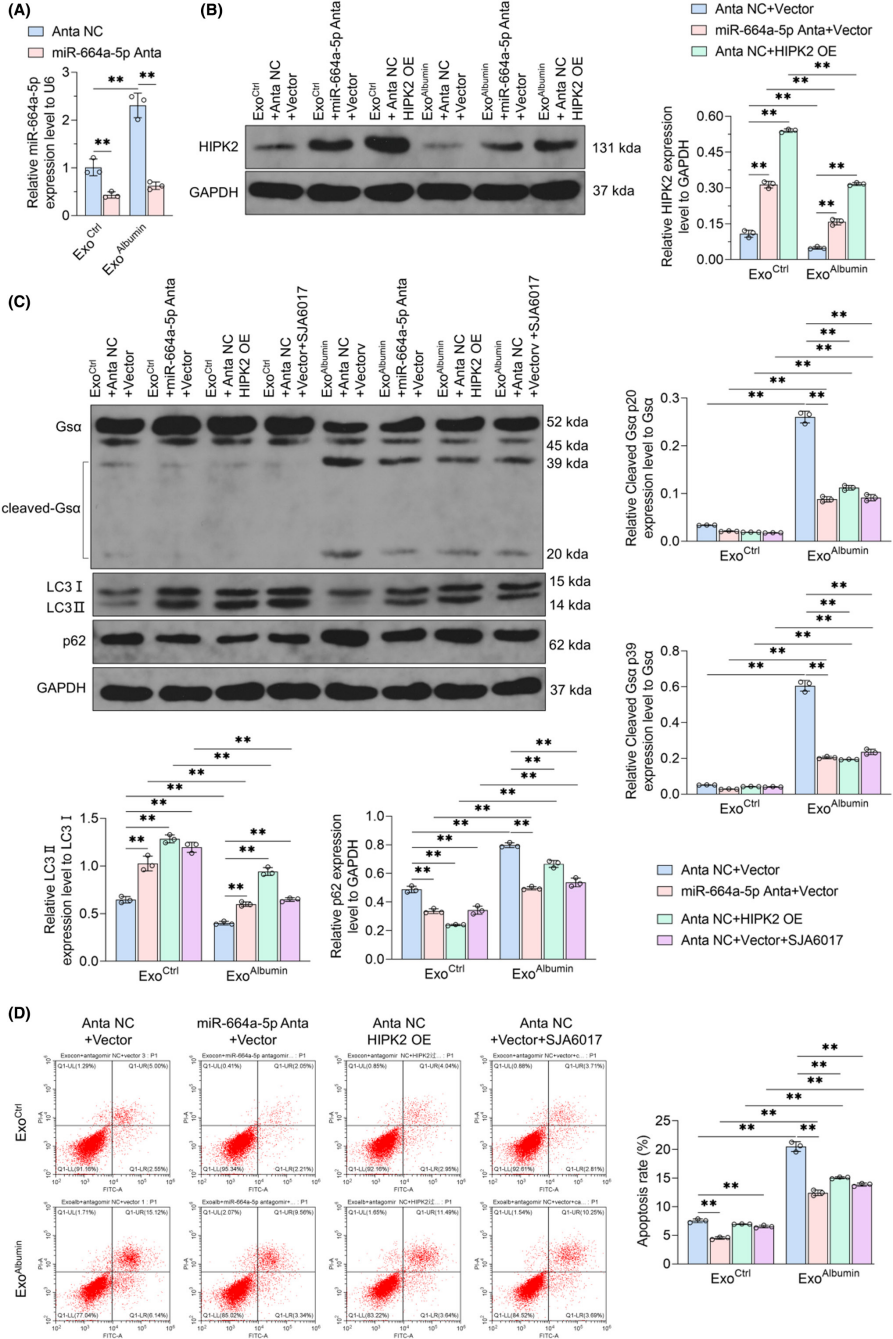
Effects of exosomes and miR‐664a‐5p antagomir, HIPK2 overexpression, and Calpain inhibitor effect on GSα shear, autophagy level, and apoptosis in podocytes. (A) The content of miR‐664a‐5p in podocytes treated with Exo^Ctrl^ or Exo^Albumin^ and combined with miRNA antagomir NC or miR‐664a‐5p antagomir for 72 h was detected by qRT‐PCR, with U6 as the internal reference. (B) Podocytes were treated with miRNA antagomir NC or miR‐664a‐5p antagomir or vector or HIPK2 overexpression plasmid, while being treated with Exo^Ctrl^ or Exo^Albumin^, respectively. Intracellular HIPK2 protein content was detected by WB after 72 h, using GAPDH as internal reference (top panel). Right is the statistical result of grey value of WB strip. (C, D) Human podocytes were treated with Exo^Ctrl^ or Exo^Albumin^ combined with miR‐664a‐5p antagomir or HIPK2 overexpression or Calpain inhibitor, respectively for 72 h. (C) GSα shear, LC3II/I ratio and p62 protein content in each group were detected by Western Blot, using GAPDH as the internal reference. (D) The apoptosis level of podocytes in each group was detected by flow cytometry with Annexin V‐FITC/PI dye. Data were expressed as Mean ± SD. One‐way analysis of variance was used for statistical analysis between groups, with ** indicating *p* < 0.01.

To determine whether renal tubular epithelial cell‐derived exosomes can induce podocyte injury through miR‐664a‐5p/HIPK2/Calpain1/GSα/autophagy pathway, podocytes were treated with miR‐664a‐5p antagomir, HIPK2 overexpression or calpain inhibitor combined with exosomes. Data showed that the promotion of Exo^Albumin^ on the expression of miR‐664a‐5p in podocytes was blocked by miR‐664a‐5p antagomir (Figure 5A). Exo^Albumin^ reduced the HIPK2 expression and this effect was eliminated by miR‐664a‐5p antagomir and HIPK2 overexpression (Figure 5B). Compared with Exo^Ctrl^, Exo^Albumin^ treatment induced GSα shear and autophagy inhibition in podocytes (Figure 5C). miR‐664a‐5p antagomir, HIPK2 overexpression plasmid, and calpain inhibitor SJA6017, all blocked the intracellular GSα shear and autophagy down‐regulation induced by Exo^Albumin^ (Figure 5C). Then, the apoptosis of podocytes was analysed. Data showed a significant increase in podocyte apoptosis after Exo^Albumin^ treatment compared with Exo^Ctrl^‐treated cells (Figure 5D). Both miR‐664a‐5p antagomir and SJA6017 reduced the apoptosis of podocytes in the Exo^Ctrl^ group, and blocked the increased apoptosis induced by Exo^Albumin^ (Figure 5D). Additionally, although HIPK2 overexpression could not change the apoptosis of podocytes treated with Exo^Ctrl^, it reduced the apoptosis of podocytes treated with Exo^Albumin^ (Figure 5D). It demonstrated that exosomes from albumin‐treated renal tubular epithelial cells could inhibit autophagy level and induce apoptosis of podocytes, depending on miR‐664a‐5p mediated HIPK2/Calpain1/GSα signal pathway inhibition.

### 3.6 Renal tubular epithelial cells‐derived exosomes enhance MN progression through miR‐664a‐5p

**FIGURE 6:**
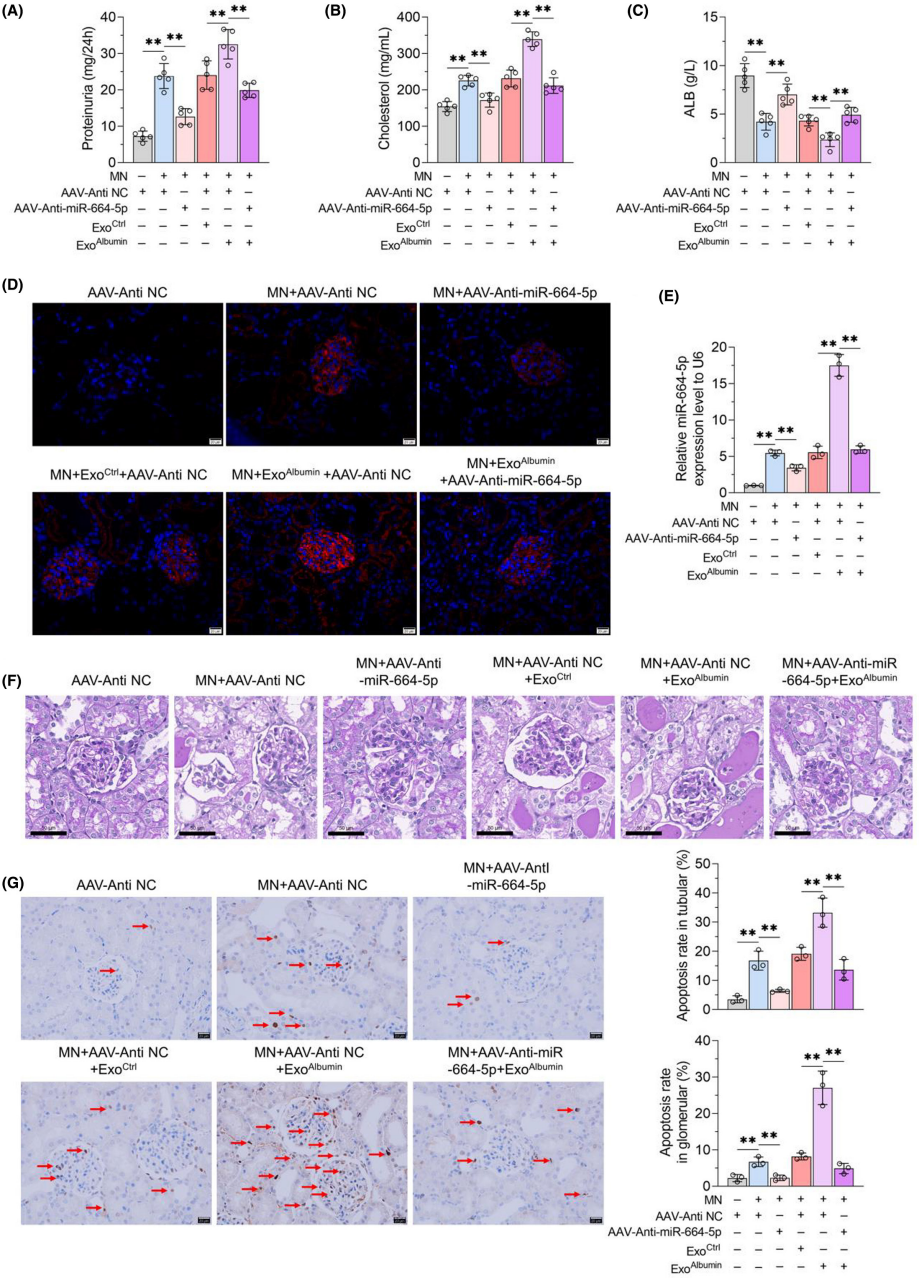
Effects of exosomes and miR‐664‐5p on MN progression and renal structural lesions in experimental MN mice. AAV‐Anti NC or AAV‐Anti‐miR‐664‐5p together with exosomes were injected into control mice or MN mice. (A) 24 h urine protein content was detected by urine protein quantitative test box. (B, C) Serum cholesterol and albumin contents of mice in each group were detected by kits. (D) Kidney tissues of control mice and MN mice were taken after different treatments, and IgG deposition was detected by IF method. Scale = 20 μm. (E) The content of miR‐664‐5p in kidney tissues of mice in each group was detected by qRT‐PCR, with U6 as the internal reference. (F) PAS method was used to observe the pathological changes of kidney tissues. Scale = 50 μm. (G) Kidney cell apoptosis was detected by TUNEL kit. Scale = 20 μm. Data were expressed as Mean ± SD. One‐way analysis of variance was used for statistical analysis between groups, with ** indicating *p* < 0.01.

**FIGURE 7:**
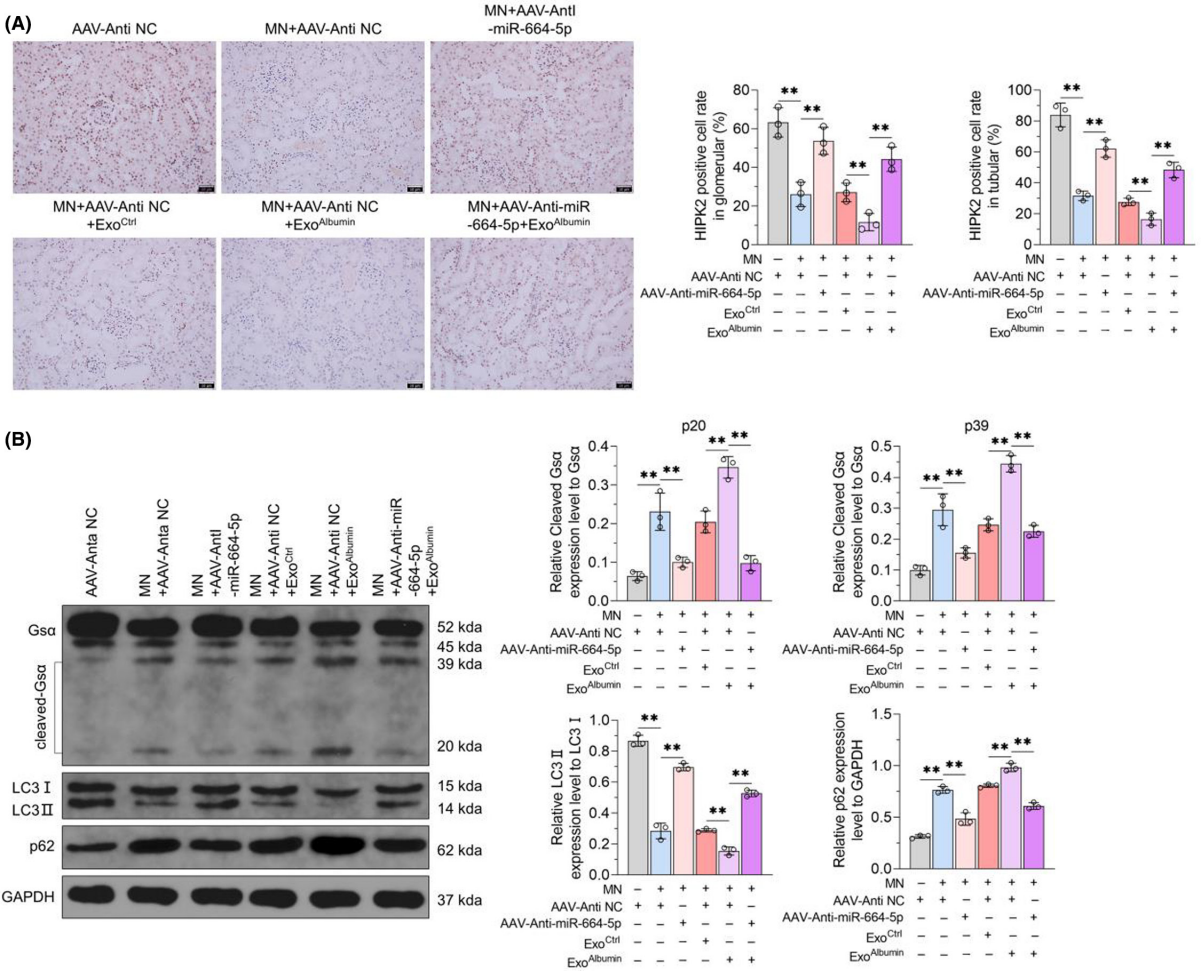
Effects of exosomes and miR‐664a‐5p on HIPK2 expression, GSα shear and autophagy in kidney tissues of experimental MN mice. (A) After injection of solvent or Exo^Ctrl^ or Exo^Albumin^ together with AAV‐Anti NC or AAV‐Anti‐miR‐664‐5p, IHC method was used to detect HIPK2 expression in kidney tissues of control mice or MN mice. Scale = 50 μm. (B) GSα shear, LC3II/I ratio and p62 protein content in kidney tissues of mice in each group were detected by Western blot, with GAPDH as the internal reference (left panel). The statistical results of grey value of WB strip are shown on the right. Data were expressed as Mean ± SD. One‐way analysis of variance was used for statistical analysis between groups, with ** indicating *p* < 0.01.

In order to explore the effects of renal tubular epithelial cells‐derived exosomes on MN progression, we constructed a mouse model of experimental MN and treated it with intravenous injection of normal saline or Exo^Ctrl^ or Exo^Albumin^. It was found that AAV‐Anti‐miR‐664‐5p treatment could ameliorate 24 h urine protein, serum cholesterol content and serum albumin down‐regulation caused by MN induction, and block the effects of Exo^Albumin^ on albuminuria and serum cholesterol and albumin content in MN mice (Figure 6A–C). Furthermore, AAV‐Anti‐miR‐664‐5p treatment could inhibit IgG deposition in glomerular of kidney tissues in MN mice (Figure 6D). While Exo^Albumin^ increased IgG fluorescence intensity in glomerular of kidney tissues in MN mice, which could be inhibited by AAV‐Anti‐miR‐664‐5p treatment (Figure 6D). And qRT‐PCR assay confirmed that compared with control mice, the expression of miR‐664a‐5p increased in kidney tissues of MN mice, which could be enhanced by Exo^Albumin^ treatment and blocked by injection of AAV‐Anti‐miR‐664‐5p (Figure 6E). Additionly, PAS staining and TUNEL assay results showed that AAV‐Anti‐miR‐664‐5p injection could improve renal tubular injury, including tubular atrophy, dilatation and vacuolation and the thickening of basement membrane in the glomeruli, and cell apotosis in tubular and in glomerular of MN mice (Figure 6F,G). However, the structural damage of renal tubules and cell apoptosis in tubular and in glomerular of MN mice, could be enhanced by Exo^Albumin^ but not Exo^Ctrl^ treatment, which were blocked by AAV‐Anti‐miR‐664‐5p (Figure 6F,G). Furthermore, MN induction could decrease HIPK2 content in renal tubule and glomerular and promote GSα shear and inhibit autophagy level of kidney of MN mice (Figure 7). Exo^Albumin^ treatment inhibited the expression of HIPK2, increased GSα shear and autophagy down‐regulation in MN mice, which could be reversed by AAV‐Anti‐miR‐664‐5p (Figure 7). It indicated that exosomes from albumin‐treated renal tubular epithelial cells, could induce cells injury of renal tubular epithelial cells and podocytes and induce HIPK2/Calpain1/GSα signal pathway‐mediated autophagy inhibition dependently of miR‐664a‐5p, leading to the progression of MN.

## 4 DISCUSSION

We previously found that hsa‐miR‐664a‐5p were specifically expressed in the urine exosomes of IMN patients but not in those of healthy volunteers.^14^ It is reported that miR‐664‐5p can regulate vascular smooth muscle cell homeostasis^34^ and promote the osteogenic differentiation ability of bone marrow mesenchymal stem cells.^35^ The homologue of hsa‐miR‐664a‐5p, namely mmu‐miR‐664‐5p, was expressed in mice, which could affect differentiation of mouse myoblasts^36^ and improve ovarian granulocell apoptosis.^37^ However, there are few reports on the correlation between miR‐664a‐5p or miR‐664‐5p and kidney. Herein, we found that the expression of miR‐664‐5p or miR‐664a‐5p increased in the kidney of experimental MN mice and in renal tubular epithelial cells exposed to albumin rather than podocytes. And albumin‐mediated renal tubular epithelial cell apoptosis was blocked by miR‐664a‐5p antagomir. AAV‐Anti‐miR‐664‐5p improved renal tubular injury and MN progression in mice. These results indicated that miR‐664a‐5p could promote MN progression by inducing apoptosis of renal tubular epithelial cells and targeted inhibition of miR‐664a‐5p improved MN process.

miRNA guides the silencing complex to degrade mRNA or hinder its translation by pairing with the target mRNA. It is reported that miR‐664a‐5p or miR‐664‐5p can bind to the mRNA of OPA1,^34^ HMGA2^35^ and p53^37^ to regulate their expression. Herein, we found that miR‐664a‐5p could bind to the 3’ UTR of HIPK2 mRNA, resulting in HIPK2 expression inhibition of renal tubules in MN mice. It has been reported that HIPK2 could promote the EMT process of renal tubular epithelial cells and renal fibrosis by activating the Smad pathway, Notch pathway, NF‐kB pathway and Wnt/β‐catenin pathway.^15^, ^16^, ^17^ In addition, HIPK2 has different apoptosis regulation effects in different diseases. Inhibition of HIPK2 expression or activation, could significantly reduce the apoptosis level of neurons treated with sevoflurane^38^ and normal rat kidney‐52E (NRK‐52E) cells with hypoxia/reoxygenation^19^ and improve myocardial apoptosis.^39^ But it is also reported that overexpression of HIPK2 can improve myocardial apoptosis induced by hypoxia reoxygenation.^40^ Downregulation of the HIPK2 inactivator SIAH1 significantly ameliorates temozolomide‐induced glioblastoma cell apoptosis.^39^ However, the role of HIPK2 in albumin‐induced renal tubular epithelial cell apoptosis is unknown. Interestingly, we found that HIPK2 knockdown could block the improving function of miR‐664a‐5p antagomir on albumin‐induced tubular epithelial cell injury, while HIPK2 overexpression could inhibit albumin‐induced tubular epithelial cell injury. It was speculated that HIPK2 plays a protective role in the apoptosis of renal tubular epithelial cells induced by albumin. indicating that HIPK2 played different apoptotic regulatory functions in different cells with different diseases. Additionally, whether HIPK2 plays a different role in the fibrosis process of renal tubular epithelial cells induced by albumin remains to be investigated.

Autophagy plays a protective role in albumin‐induced renal tubular epithelial cell injury.^32^ We focused on the role of autophagy in nephropathy for a long time, and many reports have been published, which revealed the protective effects of autophagy in diabetic nephropathy,^12^ MN,^24^ IgA nephropathy^41^ and puromycin amino nucleoside induced nephropathy.^27^ It is reported that HIPK2 enhances autophagy in hepatocellular carcinoma cells,^42^ primary hepatocytes,^25^ and spinal cord injury tissues.^43^ HIPK2 reduces the expression of Calpain1, which can shear and activate GSα, resulting in autophagy inhibition in vivo.^30^, ^31^ Here, we found that miR‐664a‐5p antagomir could block the expression of Calpain1, GSα shear and autophagy inhibition in renal tubular epithelial cells caused by albumin, which was reserved by HIPK2 knock‐down. HIPK2 overexpression and Calpain inhibitor SJA6017 could retard autophagy inhibition and apoptosis of renal tubular epithelial cells induced by albumin stimulation, which could be reversed by CQ. It demonstrated that miR‐664a‐5p/HIPK2/Calpain1/GSα signal pathway‐mediated autophagy inhibition was involved in albumin‐exposed renal tubular epithelial cell injury. Blocking miR‐664a‐5p/HIPK2/Calpain1/GSα/autophagy signalling cascade might improve MN.

Exosomes play an important role in cell paracrine function. It is reported that exosomes secreted by renal tubular epithelial cells can be taken up by macrophages.^13^ Some studies show that exosomes released by injured podocytes can promote apoptosis of renal tubular epithelial cells through miRNA‐424 and miR‐149,^44^ and podocyte‐derived exosomal miR‐221 can also mediate dedifferentiation of renal tubular epithelial cells in diabetic nephropathy.^45^ Herein, we found that renal tubular epithelial cell‐derived exosomal miR‐664a‐5p could be taken up by podocytes, leading to an increase to the expression of miR‐664a‐5p and cell apoptosis of podocytes. We also found that intravenous injection of Exo^Albumin^ increased MN progression and the apoptosis of podocytes and decreased HIPK2 expression in glomerular cells, which could be blocked by AAV‐Anti‐miR‐664‐5p. And Exo^Albumin^ could promote GSα shear and decrease autophagy level and induce cells apoptosis of podocytes, which could be reversed by miR‐664a‐5p antagomir, HIPK2 overexpression and Calpain inhibition. Thus, exosomes from albumin‐exposed renal tubular epithelial cells could aggravate MN progression through damaging podocytes, probably dependently of miR‐664a‐5p/HIPK2/Calpain/GSα signal pathway‐mediated autophagy inhibition. Furthermore, it was found that exosomes from albumin‐exposed renal tubular epithelial cells could enhance the cell apoptosis and decrease the expression of HIPK2 in tubular from MN mice. Studies have indicated that exosomes secreted by renal tubular epithelial cells after hypoxia treatment can be taken up by other renal tubular epithelial cells, thus playing a protective function.^46^ Whether exosomes from albumin‐exposed renal tubular epithelial cells could be uptaken by adjacent renal tubular epithelial cells and aggravate renal tubular injury, was unknown, which needs further investigation.

However, there are still some limitations in this study. First, it has not been confirmed that whether the imbalance of miR‐664a‐5p/HIPK2/Calpain1/GSα/autophagy signalling cascade existed in the kidney tissues of MN patients. Second, it is reported that renal tubular epithelial cell‐derived exosomal miR‐199a‐5p can enter the urine, resulting in abnormal levels of urinary exosomal miR‐199a‐5p in diabetic nephropathy patients.^13^ Moreover, we did not compare the content difference of urinary exosomes of MN mice and control mice. But results from our previous study showed that the urinary exosomal miR‐664a‐5p content of MN patients was higher than that of healthy volunteers,^14^ suggestting that renal tubular epithelial cell‐derived exosomal miR‐664a‐5p may enter the urine and is a potential biomarker for the diagnosis of MN, which needs to be verified by clinical trials with large‐sample size samples.

## 5 CONCLUSION

**FIGURE 8:**
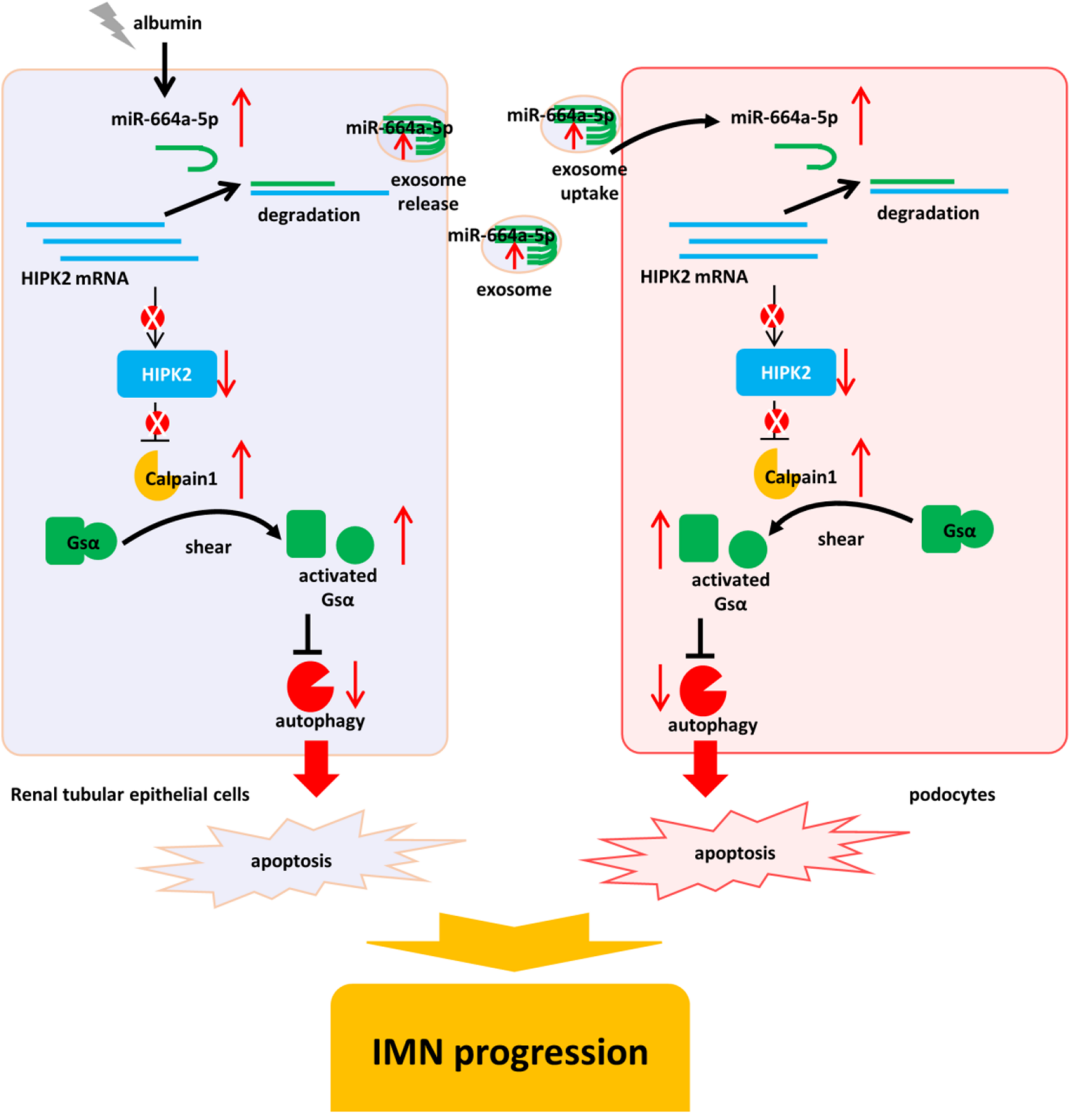
Schematic diagram of albumin‐mediated miR‐664a‐5p upregulation causing renal tubular epithelial cells injury and podocytes damage via exosome through HIPK2/Calpain/GSα‐ mediated autophagy inhibition.

In this investigation, albumin could increase the content of miR‐664a‐5p in renal tubular epithelial cells, resulting in renal tubular epithelial cell injury and triggering MN progression through HIPK2/Calpain1/GSα signalling pathway‐mediated autophagy inhibition. Meanwhile, we found that miR‐664a‐5p in renal tubular epithelial cells can be transferred horizontally into podocytes through exosomes, leading to the imbalance of miR‐664a‐5p/HIPK2/Calpain1/GSα/autophagy signalling cascade in podocytes, inducing podocytes apoptosis and aggravating MN progression. Inhibition of miR‐664a‐5p/HIPK2/Calpain1/GSα/autophagy signalling cascade can reverse the above process and improve the MN process, thus becoming a potential therapeutic target for MN. The final scheme was summarized in Figure 8.

## AUTHOR CONTRIBUTIONS

**Zhiming Shan:** Conceptualization (equal); investigation (equal); methodology (equal); writing – original draft (equal). **Zhenchao Zhuang:** Conceptualization (equal); investigation (equal); methodology (equal); writing – original draft (equal). **Peiyao Ren:** Methodology (equal); software (equal). **Li Zhao:** Data curation (equal); investigation (equal); software (equal); validation (equal). **Danna Zheng:** Formal analysis (equal); resources (equal); validation (equal). **Wei Chen:** Project administration (equal); validation (equal); visualization (equal); writing – review and editing (equal). **Juan Jin:** Funding acquisition (equal); project administration (equal); supervision (equal); visualization (equal); writing – review and editing (equal).

## FUNDING INFORMATION

This research was supported by the Huadong Medicine Joint Funds of the Zhejiang Provincial Natural Science Foundation of China (Grant no. LHDMZ22H050001); The Construction of Key Projects by Zhejiang Provincial Ministry (Project no. WKJ‐ZJ‐2302); The Key Project of Scientific Research Foundation of Chinese Medicine (2022ZZ002); the ‘Pioneer’ and ‘Leading Goose’ R&D Program of Zhejiang (2023C03075); The Key project of Basic Scientific Research Operating Funds of Hangzhou Medical College (KYZD202002); Science Research Fund Project of Zhejiang Chinese Medical University (2020ZG37,2022FSYYZY01).

## CONFLICT OF INTEREST STATEMENT

The authors confirm that there are no conflicts of interest.

## Supporting information


Figure S1.

## Data Availability

The data sets used or analysed during this study are available from the corresponding author upon reasonable request.
