# Supplementary material for: miR‐664a‐5p promotes experimental membranous nephropathy progression through HIPK2/Calpain1/GSα‐mediated autophagy inhibition
Source: J Cell Mol Med. 2024 Jan 7;28(3):e18074. doi: 10.1111/jcmm.18074 (PMC10844711; doi:10.1111/jcmm.18074)
Supplement: Supplementary file 1 — Figure S1. [file JCMM-28-e18074-s001.docx]

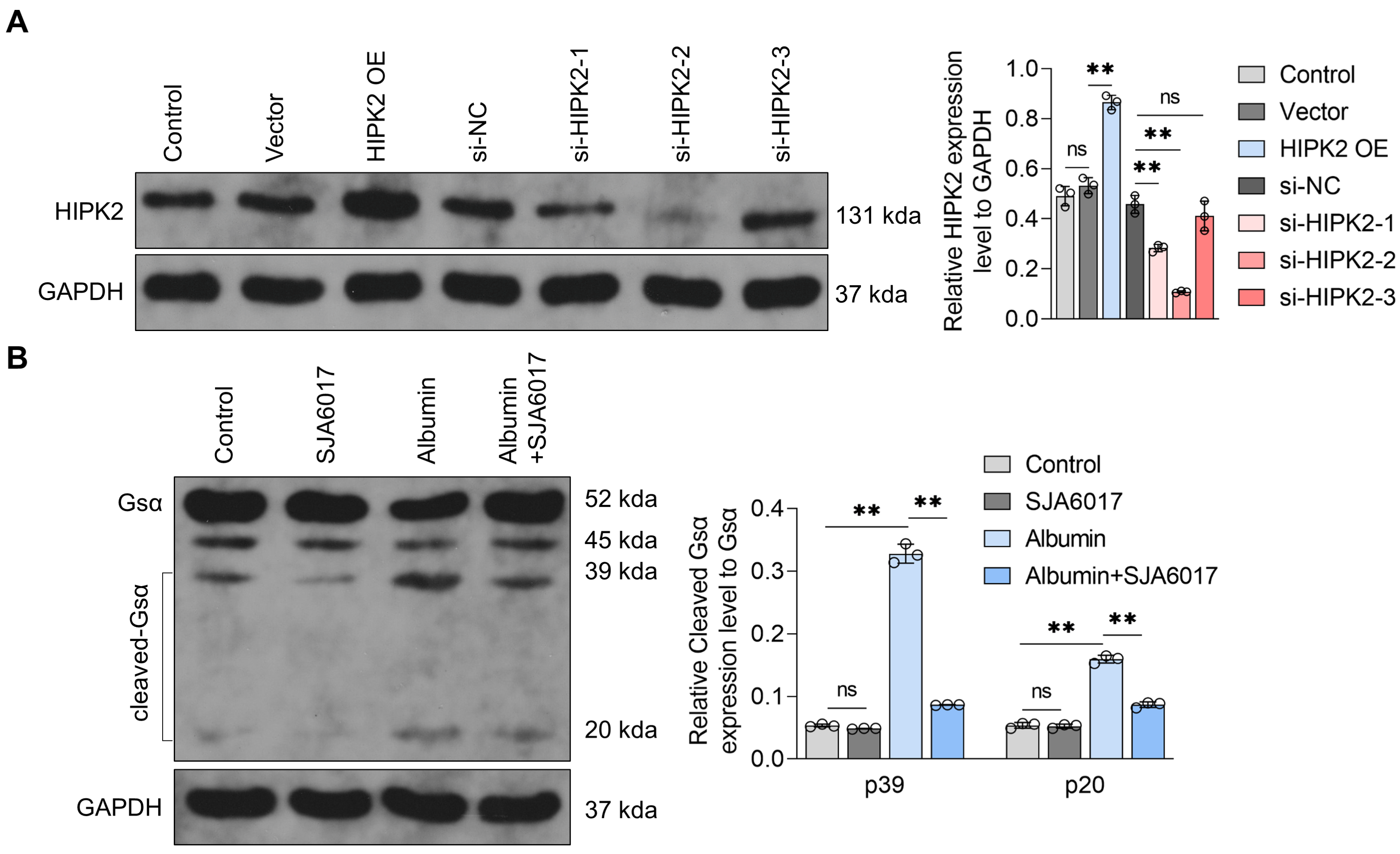


**Supplementary Figure 1.** Effects of HIPK2 overexpression and knock-down treatment on HIPK2 expression and effects of Calpain inhibitor on Gsα shear in HK-2 cells. (A) WB was used to detect the HIPK2 protein content in HK-2 cells after 72h treatment of the vector or HIPK2 overexpression plasmid or NC siRNA or HIPK2 siRNA, using GAPDH as the internal reference. Statistical results of gray values of WB strips in the right. (B) Western Blot analysis of GSα shear in HK-2 cells treated with solvent or SJA6017 and albumin for 72h, using GAPDH as internal reference. Statistical results of gray values of WB strips in the right. Data were expressed as Mean±SD. One-way analysis of variance was used for statistical analysis between groups, with ** indicating p<0.01 and ns indicating p>0.05.
